# IL-15 Promotes the Survival of Anti-Inflammatory (M2), Immunoinhibitory (IL-10+) Dermal Macrophages in Human Eyelid Skin Under IFNγ-Dominated Inflammatory Conditions

**DOI:** 10.3390/ijms26167811

**Published:** 2025-08-13

**Authors:** Dana-Lee Demetrius, Sofia M. Perez, Takahiro Suzuki, Jennifer Gherardini, Wendy Lee, Jérémy Chéret, Ralf Paus

**Affiliations:** 1 Dr. Phillip Frost Department of Dermatology & Cutaneous Surgery, University of Miami Miller School of Medicine, Miami, FL 33125, USA; dxb1181@miami.edu (D.-L.D.); smp254@med.miami.edu (S.M.P.); dermatakalogy@gmail.com (T.S.); j.gherardini@cutaneon.com (J.G.); rxp803@med.miami.edu (R.P.); 2 Department of Dermatology, Hamamatsu University, Hamamatsu 431-3192, Japan; 3 CUTANEON–Skin & Hair Innovations GmbH, 22335 Hamburg, Germany; 4 Bascom Palmer Eye Institute, University of Miami Miller School of Medicine, Miami, FL 33125, USA; wlee@med.miami.edu

**Keywords:** IL-15, IFNγ, macrophages, human skin, inflammation, skin diseases, psoriasis, atopic dermatitis

## Abstract

Interleukin (IL)-15 is primarily known as a pro-inflammatory and anti-apoptotic cytokine, which stimulates the proliferation and survival of key immunocytes, including macrophages (MACs). Yet, it remains unclear how IL-15 specifically impacts MACs in intact human skin, particularly immunoinhibitory, IL-10-producing/secreting M2 MACs (CD206^+^IL-10^+^). In the current pilot study, we explored this in organ-cultured healthy human eyelid skin in the presence of IFNγ (100 IU/mL) to mimic a pro-inflammatory signaling milieu found in several chronic immunodermatoses. Quantitative immunohistomorphometry showed that IFNγ significantly reduced the number of CD68^+^MACs, M2 CD206^+^MACs, and immunoinhibitory CD206^+^IL-10^+^MACs. Moreover, co-administering recombinant human (rh) IL-15 after inducing inflammation by IFNγ largely reversed the IFNγ-induced decline in MAC populations. To investigate if this was mediated via the private IL-15 receptor alpha (IL-15Rα), we successfully silenced IL-15Rα in human skin ex vivo. Indeed, co-administration of IL-15Rα siRNA abrogated the rhIL-15 protection of M2 CD206^+^MACs against IFNγ, but not of the CD206^+^IL-10^+^MAC subpopulation. These pilot data suggest that IL-15 maintains immunoinhibitory M2 CD206^+^IL-10^+^MACs in human skin under IFNγ-dominated inflammatory conditions. Therefore, it deserves to be explored whether IL-15 or IL-15Rα agonists can exert therapeutic benefit in chronic inflammatory dermatoses by preserving the intracutaneous pool of anti-inflammatory dermal M2 MACs.

## 1. Introduction

Macrophages (MACs) are the most abundant and functionally important innate immune cells in the human dermis [1,2,3], which can be derived from both circulating blood monocytes [4,5] and tissue-resident progenitor cells in human skin [6]. MAC plasticity allows classically activated ΜACs to switch from a pro-inflammatory phenotype (M1), capable of stimulating a Th1 immune response [4,7] to an anti-inflammatory phenotype (M2), capable of stimulating a Th2 immune response and secreting immunoinhibitory cytokines like IL-10 that are critical for tissue repair [8] and inflammation suppression [7,9,10]. Given their nearly opposite roles and influence on cutaneous inflammation, M1 and M2 MACs exist at a tightly regulated ratio in order to maintain a balance between pro- and anti-inflammatory activity [3,5,11]. Dysfunction in this M1:M2 ratio can result in pathologic inflammatory responses and chronic disease development [3,4,12]. Dermal M1 and M2 MACs play an important role in developing and maintaining several chronic immunodermatoses [13,14] such as atopic dermatitis [15] and psoriasis [16,17] notably driven by the potent, pro-inflammatory cytokine IFNγ [18,19]. While pathologically elevated levels of IFNγ promote the recruitment and activation of M1 ΜACs [10,20], it is less well-understood how IFNγ impacts immunoinhibitory M2 MACs in human skin, and how IFNγ interacts with other presumably pro-inflammatory cytokines found in chronic immunodermatoses such as IL-15 [21,22].

IL-15 is a pleiotropic, anti-apoptotic, and immunomodulatory cytokine expressed and produced by, e.g., keratinocytes, fibroblasts, monocytes, and MACs in the skin [23,24,25,26]. IL-15 stimulates both the proliferation and survival of dermal ΜACs and dendritic cells [27,28]. While previous research in human tissues has primarily focused on the pro-inflammatory functions of IL-15, namely in the pathogenesis of IFNγ-driven immunodermatoses [29,30,31,32], IL-15 also exerts important anti-inflammatory activities [23,33,34,35]. For example, IL-15 operates as an immune privilege guardian in human hair follicles by maintaining the population of potently IL-10-secreting inhibitory natural killer T (iNKT10) cells in human scalp hair follicles in vivo [23] protecting them from alopecia areata development [36]. At low concentrations, IL-15 can also enhance IL-10 production from murine peritoneal MACs; this indicates a potential anti-inflammatory role mediated through the high-affinity IL-15 receptor-alpha (IL-15Rα), which is uniquely activated at low levels of IL-15 [34,35]. Yet, it remains unclear how IL-15 impacts MACs, namely anti-inflammatory, IL-10-producing/secreting M2 MACs (CD206^+^IL-10^+^), in intact human skin under pro-inflammatory conditions [2,3,4,31].

The current pilot study addresses the open question of whether IL-15 signaling can regulate the overall number of (a) CD68^+^MACs, (b) M1 (CD68^+^CD86^+^)/M2 (CD206^+^) MAC ratio [2,37,38] and (c) immunoinhibitory M2 MACs (CD206^+^IL-10^+^) under IFNγ-dominated inflammatory human skin organ culture conditions that experimentally mimic chronic immunodermatoses ex vivo. Also, we asked which of the observed MAC effects of IL-15 were mediated by the private IL-15Rα subunit [27,28].

## 2. Results

### 2.1. IFNγ Reduces the Total Pool of Human Dermal MACs Ex Vivo

**Figure 1:**
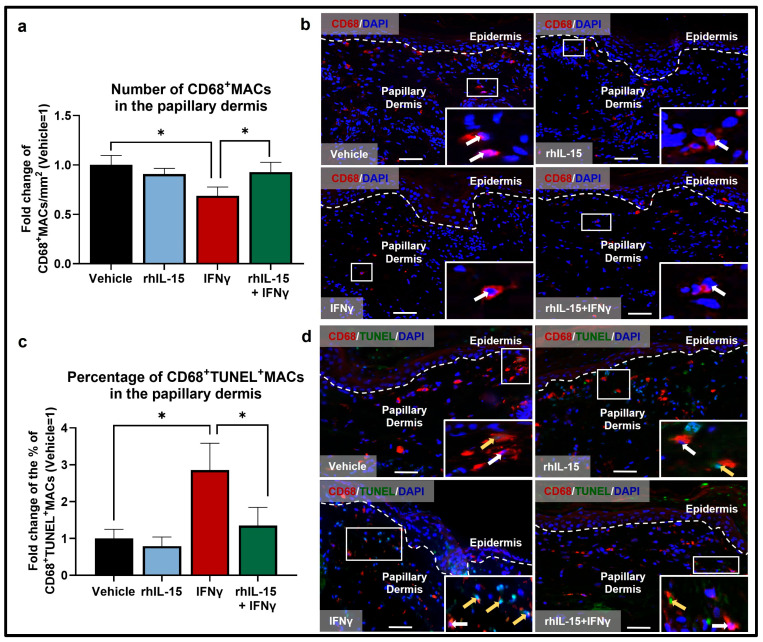
IFNγ reduces the pool of human dermal CD68^+^MACs, while co-administration of rhIL-15 protects against IFNγ-induced CD68^+^MAC apoptosis in the papillary dermis of human skin ex vivo. (**a**) Quantitative analysis of CD68^+^MACs. N = 12–14 skin sections from 4 independent donors treated with vehicle, 100 ng/mL rhIL-15, 100 IU/mL IFNγ, or 100 ng/mL of rhIL-15 + 100 IU/mL IFNγ. (**b**) Representative fluorescence images of CD68 immunofluorescence. (**c**) Quantitative analysis of CD68^+^TUNEL^+^MACs. N = 9–11 skin sections from 3 independent donors treated with vehicle, 100 ng/mL rhIL-15, 100 IU/mL IFNγ, or 100 ng/mL of rhIL-15 + 100 IU/mL IFNγ. (**d**) Representative fluorescence images of CD68 and TUNEL double immunofluorescence. Analyses were performed in defined reference areas in the papillary dermis, 200 µm below the dermal-epidermal junction [6]. Fold change in Mean ± SEM, unpaired Student’s *t*-test or Mann–Whitney U-test, * *p* < 0.05, two-tailed. Scale bar: 50 µm. Nuclei counterstained with DAPI (blue). White hatched lines indicate epidermal basement membrane. White arrows indicate CD68^+^MACs. Yellow arrows indicate CD68^+^TUNEL^+^MACs. For the bar graph, black, blue, red, and green bars represent data from skin treated with vehicle, rhIL-15, IFNγ, and rhIL-15+IFNγ, respectively.

First, we investigated how recombinant human (rh) IL-15 (100 ng/mL) or IFNγ (100 IU/mL) independently impact the overall number of dermal MACs ex vivo. After 6 days of treatment, quantitative immunohistomorphometry (qIHM) showed that, compared to vehicle-treated control skin, IFNγ significantly reduced the number of interfollicular CD68^+^MACs in the papillary dermis (Figure 1a,b). This is most likely occurring by the promotion of MAC apoptosis by IFNγ, as indicated by the significantly increased percentage of CD68^+^MACs undergoing apoptosis (TUNEL^+^) in IFNγ-treated skin compared to vehicle-treated control skin (Figure 1c,d).

**Figure 2:**
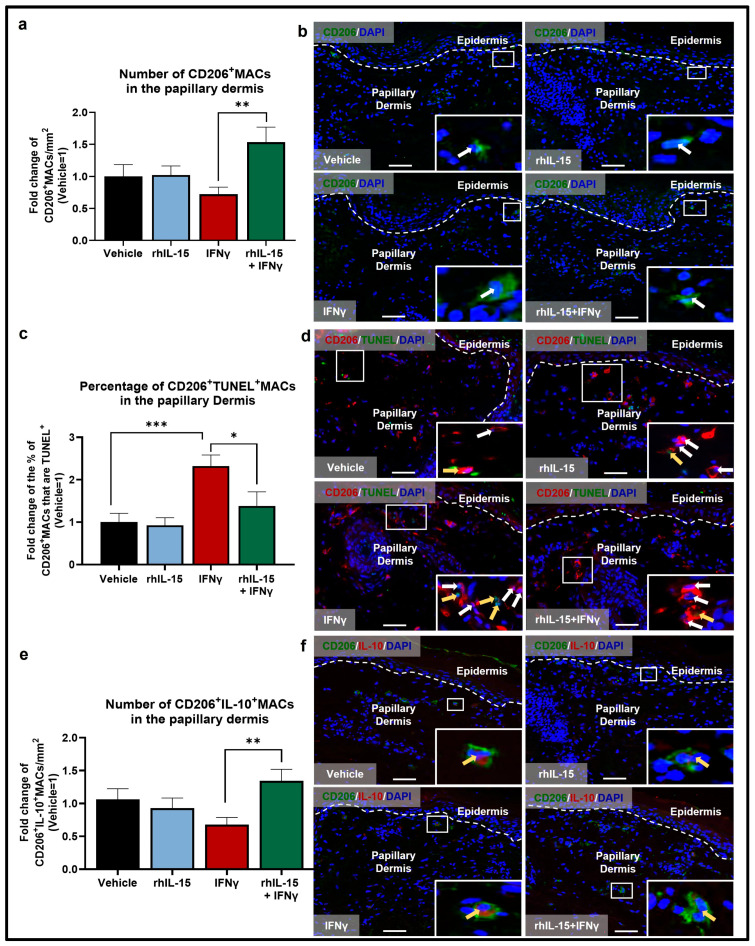
IFNγ reduces the pool of dermal CD206^+^MACs by promoting their apoptosis, while co-administration of rhIL-15 significantly restores the number of CD206^+^MACs and CD206^+^IL-10^+^MACs in the papillary dermis of human skin ex vivo. (**a**) Quantitative analysis of CD206^+^MACs. N = 14–16 skin sections from 4 independent donors treated with vehicle, 100 ng/mL rhIL-15, 100 IU/mL IFNγ, or 100 ng/mL of rhIL-15 + 100 IU/mL IFNγ. (**b**) Representative fluorescence images of CD206 immunofluorescence. (**c**) Quantitative analysis of CD206^+^TUNEL^+^MACs. N = 13 skin sections from 4 independent donors treated with vehicle, 100 ng/mL rhIL-15, 100 IU/mL IFNγ, or 100 ng/mL of rhIL-15 + 100 IU/mL IFNγ. (**d**) Representative fluorescence images of CD206 and TUNEL double immunofluorescence. (**e**) Quantitative analysis of CD206^+^IL-10^+^MACs. N = 14–15 skin sections from 4 independent donors treated with vehicle, 100 ng/mL rhIL-15, 100 IU/mL IFNγ, or 100 ng/mL of rhIL-15 + 100 IU/mL IFNγ. (**f**) Representative fluorescence images of CD206 and IL-10 double immunofluorescence. Analyses were performed in defined reference areas in the papillary dermis, 200 µm below the dermal-epidermal junction [6]. Fold change in Mean ± SEM, unpaired Student’s *t*-test or Mann–Whitney U-test, * *p* < 0.05, ** *p* < 0.01, *** *p* < 0.001, two-tailed. Scale bar: 50 µm. Nuclei counterstained with DAPI (blue). White hatched lines indicate epidermal basement membrane. White arrows indicate CD206^+^MACs. Yellow arrows indicate CD206^+^IL-10^+^MACs or CD206^+^TUNEL^+^MACs. For the bar graph, black, blue, red, and green bars represent data from skin treated with vehicle, rhIL-15, IFNγ, and rhIL-15+IFNγ, respectively.

**Figure 3:**
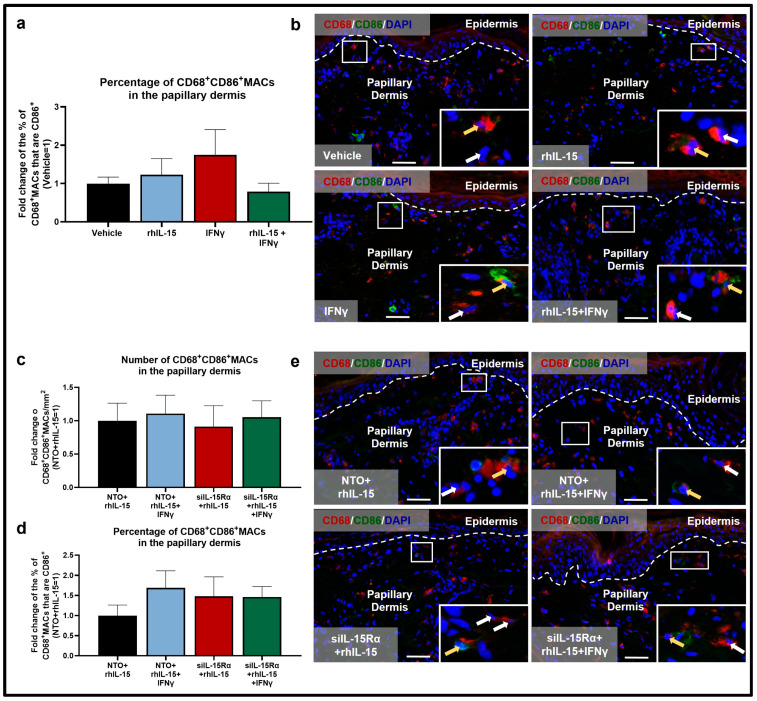
IFNγ does not significantly influence the population of M1 CD68^+^CD86^+^MACs. (**a**) Quantitative analysis of M1 CD68^+^CD86^+^MACs. N = 12–15 skin sections from 4 independent donors treated with vehicle, 100 ng/mL rhIL-15, 100 IU/mL IFNγ, or 100 ng/mL of rhIL-15 + 100 IU/mL IFNγ. (**b**) Representative fluorescence images of CD68 and CD86 immunofluorescence. (**c**,**d**) Quantitative analysis of M1 CD68^+^CD86^+^MACs. N = 11–12 skin sections from 3 independent donors treated with vehicle, 100 ng/mL rhIL-15, 100 IU/mL IFNγ, or 100 ng/mL of rhIL-15 + 100 IU/mL IFNγ. (**e**) Representative fluorescence images of CD68 and CD86 immunofluorescence. Analyses were performed in defined reference areas in the papillary dermis, 200 µm below the dermal-epidermal junction [6]. Fold change in Mean ± SEM, unpaired Student’s *t*-test or Mann–Whitney U-test, not significant, two-tailed. Scale bar: 50 µm. Nuclei counterstained with DAPI (blue). White hatched lines indicate epidermal basement membrane. White arrows indicate CD68^+^MACs. Yellow arrows indicate M1 CD68^+^CD86^+^MACs. For the bar graph, black, blue, red, and green represent data from skin treated with NTO+rhIL-15, NTO+rhIL-15+IFNγ, siIL-15Rα+rhIL-15, siIL-15Rα+rhIL-15+IFNγ, respectively.

Although not significant, IFNγ also slightly reduced the number of M2 CD206^+^MACs (Figure 2a,b), again by promoting apoptosis, as demonstrated by a significant increase in the percentage of M2 CD206^+^TUNEL^+^MACs in IFNγ-treated skin compared to control (Figure 2c,d). This result also confirmed the anti-apoptotic properties of IL-15, in this case for protecting anti-inflammatory M2 MACs from IFNγ-induced apoptosis. Also, IFNγ-treated skin showed a reduced percentage of immunoinhibitory CD206^+^IL-10^+^MACs compared to vehicle-treated skin (Figure 2e,f). Finally, as expected, IFNγ increased the percentage of M1 CD68^+^CD86^+^MACs, although this was not statistically significant (Figure 3a,b).

Altogether, these data confirmed that IFNγ stimulates pro-inflammatory M1 MACs to the detriment of anti-inflammatory M2 MACs. To our knowledge, this constitutes the first evidence that IFNγ promotes apoptosis of native dermal CD68^+^MACs in general and of immunoinhibitory CD206^+^IL-10^+^MACs under clinically relevant ex vivo conditions in human skin and that rhIL-15 may rescue immunoinhibitory CD206^+^IL-10^+^MACs under inflammatory IFNγ-dominated conditions.

Conversely, rhIL-15 alone did not change any of the above MAC read-outs in the absence of high-level IFNγ (Figure 1, Figure 2 and Figure 3) suggesting that IL-15’s anti-inflammatory effect on dermal MACs may depend on the surrounding tissue immune-environment and levels of other cytokines in particular, like IFNγ.

### 2.2. rhIL-15 Reverses the IFNγ-Induced Reduction in M2 CD206^+^MACs

However, administration of 100 ng/mL rhIL-15 after 48 h of IFNγ exposure significantly reversed the decrease in total number of interfollicular dermal CD68^+^MACs (Figure 1a,b) and significantly reduced the percentage of apoptotic CD68^+^TUNEL^+^MACs (Figure 1c,d). Importantly, rhIL-15 restored the number of M2 CD206^+^MACs (Figure 2a,b) by significantly reducing the percentage of CD206^+^MACs undergoing apoptosis (Figure 2c,d) compared to skin treated with IFNγ alone. rhIL-15 also significantly restored the subset of immunoinhibitory M2 CD206^+^IL-10^+^MACs (Figure 2e,f). Furthermore, the co-administration of rhIL-15 with IFNγ, reverses the increased percentage of M1 CD68^+^CD86^+^MACs, though this was not statistically significant (Figure 3a,b). Overall, our data showed that in an IFNγ-dominated skin milieu, rhIL-15 tended to expand or preserve the population of both M2 CD206^+^MACs and M2 CD206^+^IL-10^+^MACs. Thus, rhIL-15 can protect M2 CD206^+^MACs and their immunoinhibitory (IL-10 secreting) subpopulation from IFNγ-induced apoptosis, possibly through upregulation of anti-apoptotic factors such as Bcl-2 and Bcl-xL [28,39].

### 2.3. Human Dermal M2 CD206^+^MAC Survival Depends on IL-15Rα-Mediated Signaling

**Figure 4:**
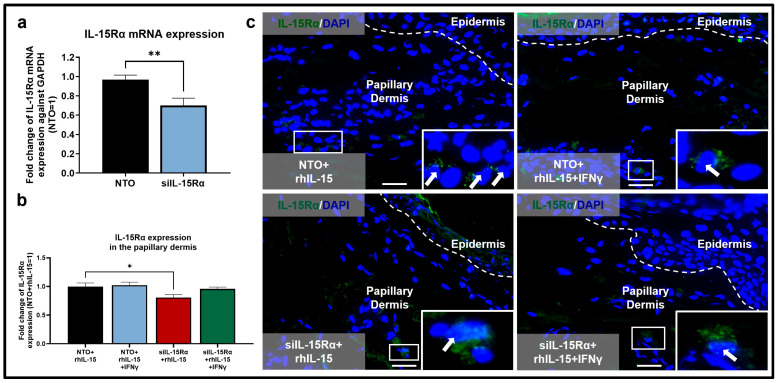
Successful IL-15Rα knock-down in human skin was demonstrated by decreased levels of IL-15Rα mRNA and protein expression in the papillary dermis of human skin ex vivo. (**a**,**b**) Quantitative analysis of IL-15Rα (**a**) mRNA expression, N = 3–4 skin punches per group from 3 donors and (**b**) protein expression, N = 12–16 skin sections from 3 independent donors treated with siRNA for IL-15Rα or non-targeting oligos (NTO) in the presence of 100 ng/mL rhIL-15, 100 IU/mL IFNγ, or 100 ng/mL of rhIL-15 and 100 IU/mL IFNγ. (**c**) Representative fluorescence images of IL-15Rα immunofluorescence. Analyses were performed in defined reference areas in the papillary dermis, 200 µm from the epidermal basement membrane. Fold change in Mean ± SEM, unpaired Student’s *t*-test or Mann–Whitney U-test, * *p* < 0.05, ** *p* < 0.01, two-tailed. Scale bar = 50 µm. Nuclei counterstained with DAPI (blue). White hatched lines indicate epidermal basement membrane. White arrows indicate IL-15Rα protein expression. For the bar graph, black, blue, red, and green represent data from skin treated with NTO+rhIL-15, NTO+rhIL-15+IFNγ, siIL-15Rα+rhIL-15, siIL-15Rα+rhIL-15+IFNγ, respectively.

Next, we asked if the rhIL-15-induced MAC effects described above were mediated via specific activation of the private receptor IL-15Rα [28,40]. To do so, we successfully silenced IL-15Rα in human skin, as shown by the significantly decreased IL-15Rα mRNA and protein expression (Figure 4a,c). To the best of our knowledge, this represents the first knockdown of IL-15Rα in intact human skin ex vivo and adds an instructive new preclinical research tool for interrogating the roles of IL-15/IL-15Rα signaling in human skin and confirms the direct dependence of our results on IL-15Rα function under non-inflammatory conditions. Under inflammatory IFNγ-dominated conditions, however, IL-15Rα silencing at the protein level was not significantly reduced but decreased by around 7% (Figure 4b,c). Previously, a knockdown efficiency of around 10% at the protein level ex vivo has been shown to impact downstream IL-15 signaling [23,41]. This non-significant reduction in IL-15Rα may result from an increased half-life of IL-15Rα while bound to IL-15 only under inflammatory IFNγ-dominated conditions to extend its therapeutic properties.

**Figure 5:**
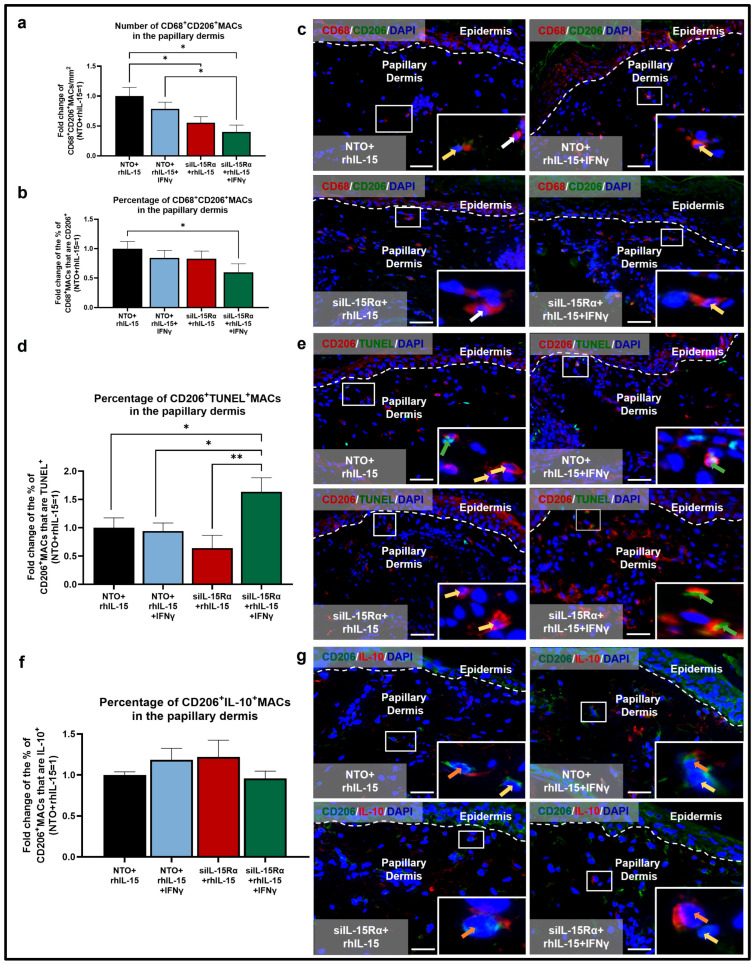
siIL-15Rα in the presence of rhIL-15 amplifies the reduced number of CD68^+^CD206^+^MACs and their apoptosis under IFNγ-induced inflammation apoptosis but does not affect CD206^+^IL-10^+^MAC levels in the papillary dermis of human skin ex vivo. (**a**,**b**) Quantitative analysis of CD68^+^CD206^+^MACs (**a**) number and (**b**) percentage. N = 16–18 skin sections per group from 3 independent donors treated with siRNA for IL-15Rα or non-targeting oligos (NTO) in the presence of 100 ng/mL rhIL-15 or 100 ng/mL of rhIL-15 + 100 IU/mL IFNγ. (**c**) Representative fluorescence images of CD68 and CD206 double immunofluorescence. (**d**) Quantitative analysis of CD206^+^TUNEL^+^MACs. N = 12–18 skin sections from 3 independent donors treated with siRNA for IL-15Rα or non-targeting oligos (NTO) in the presence of 100 ng/mL rhIL-15 or 100 ng/mL of rhIL-15 + 100 IU/mL IFNγ. Notably, some CD68^−^CD206^+^ cells can be seen, which likely represent dendritic cells [42]. (**e**) Representative fluorescence images of CD206 and TUNEL double immunofluorescence. (**f**) Quantitative analysis of CD206^+^IL-10^+^MACs. N = 10–16 skin sections from 3 independent donors treated with siRNA for IL-15Rα or non-targeting oligos (NTO) in the presence of 100 ng/mL rhIL-15 or 100 ng/mL of rhIL-15 + 100 IU/mL IFNγ. (**g**) Representative fluorescence images of CD206 and IL-10 double immunofluorescence. Analyses were performed in defined reference areas in the papillary dermis, 200 µm below the dermal-epidermal junction [6]. Fold change in Mean ± SEM, unpaired Student’s *t*-test, * *p* < 0.05, ** *p* < 0.01, two-tailed. Scale bar: 50 µm. Nuclei counterstained with DAPI (blue). White hatched lines indicate epidermal basement membrane. White arrows indicate CD68^+^MACs. Yellow arrows indicate CD68^+^CD206^+^MACs. Green arrows indicate CD206^+^TUNEL^+^MACs. Orange arrows indicate CD206^+^IL-10^+^MACs. For the bar graph, black, blue, red, and green represent data from skin treated with NTO+rhIL-15, NTO+rhIL-15+IFNγ, siIL-15Rα+rhIL-15, siIL-15Rα+rhIL-15+IFNγ, respectively.

Under non-inflammatory conditions, silencing IL-15Rα in the presence of rhIL-15 significantly reduced the number of M2 CD68^+^CD206^+^MACs compared to skin treated with non-targeting oligos (NTO)+rhIL-15 (Figure 5a,c). In contrast, the number of M1 CD68^+^CD86^+^MACs remains unaffected, but the proportion of M1 CD68^+^CD86^+^MACs among the total population of CD68^+^MACs is increased, although it did not reach statistical significance (Figure 3c,e). These data indicate that a baseline level of continuous IL-15Rα-mediated signaling is important for M2 CD68^+^CD206^+^MAC survival or differentiation under non-inflammatory, physiological conditions. Also under this condition, since the percentage of apoptotic M2 CD206^+^TUNEL^+^MACs was not increased with IL-15Rα knockdown (Figure 5d,e), this suggests that IL-15Rα is mainly required for promoting CD68^+^MAC differentiation into M2 CD68^+^CD206^+^MACs (Figure 5b) in a physiological, non-inflammatory environment.

Under pro-inflammatory IFNγ-dominated conditions, IL-15Rα signaling is required for M2 CD68^+^CD206^+^MACs survival: co-administration of IFNγ and IL-15Rα silencing in the presence of rhIL-15 significantly reduced the number and percentage (not significant) of M2 CD68^+^CD206^+^MACs compared to NTO+rhIL-15+IFNγ-treated skin (Figure 5a–c). This is likely occurring via the significant increase in M2 CD206^+^MAC apoptosis seen in the siIL-15Rα+rhIL-15+IFNγ condition (Figure 5d,e). Moreover, the proportion of M1 CD68^+^CD86^+^MACs remains unchanged between NTO+rhIL-15+IFNγ and siIL-15Rα+rhIL-15+IFNγ (Figure 3c–e). Interestingly, silencing IL-15Rα did not significantly affect the percentage of the subpopulation of CD206^+^MACs that express/secrete IL-10 (Figure 5f,g). This suggests that IL-10 production by M2 CD206^+^MACs and the (non-statistically significant) increased proportion of M1 CD68^+^CD86^+^MACs do not rely on intact IL-15Rα signaling and may instead be mediated through activation of the IL-15Rβ/γc heterodimer and/or by an effect on IFNγ signaling.

## 3. Discussion

**Figure 6:**
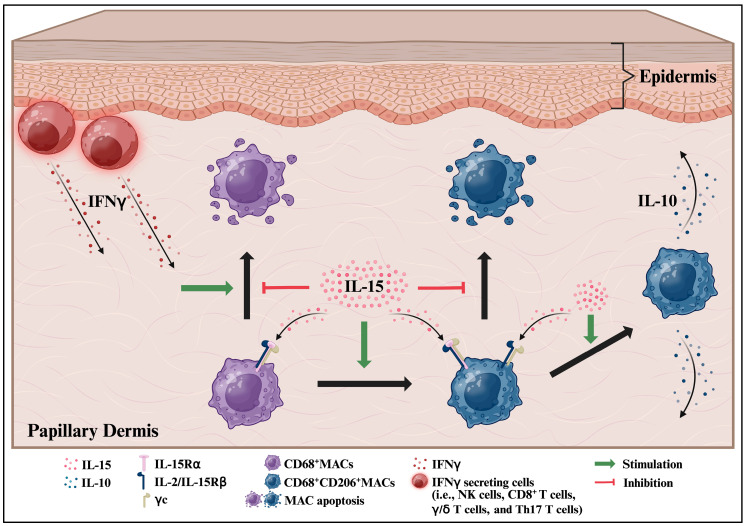
Cartoon depicting rhIL-15 effects on dermal MACs during IFNγ-driven inflammatory conditions in intact human skin ex vivo. While IFNγ promotes MAC apoptosis, IL-15/IL-15Rα mediated signaling blocks this apoptosis to maintain the pool of dermal CD68^+^MACs and CD206^+^MACs. IL-15/IL-15Rα signaling may also promote CD68^+^MACs differentiation into anti-inflammatory M2 CD68^+^CD206^+^MACs. Under IFNγ-driven inflammation, IL-15 signaling through the IL-2/IL-15Rβ and γc heterodimer may promote the production of IL-10 from M2 MACs in the papillary dermis of human skin ex vivo. Created in BioRender. Demetrius, D. ((Accessed on 5 July 2025)) https://BioRender.com/olvixfc. (Science Suite Inc. Toronto, Canada).

While IL-15 is generally portrayed as a pro-inflammatory and autoimmunity-enhancing cytokine in human skin [10,40,43], our pilot study highlights the underestimated anti-inflammatory functions of IL-15 and IL-15Rα-mediated signaling in human skin during IFNγ-mediated inflammation. rhIL-15 not only restores the population of CD68^+^MACs and M2 CD206^+^ΜACs under IFNγ-dominated inflammation in human skin but also promotes the survival and expansion of immunoinhibitory M2 CD206^+^IL-10^+^MACs (Figure 6). These observations call for much more systematic exploration and are in line with other anti-inflammatory activities we have previously shown for rhIL-15 in human skin and its appendages, namely its stimulatory effect on IL-10-secreting regulatory/immunoinhibitory NKT cells and its underappreciated role as an important hair follicle immune privilege guardian [23]. Future assays focusing on total IL-10 protein or mRNA expression in the dermis as well as pro-inflammatory cytokines (e.g., IL-1β, IL-17, TNFα) after rhIL-15 exposure under IFNγ-dominated inflammatory conditions may help to further quantify the functional immunoinhibitory properties of IL-15 on human skin MACs and their potential therapeutic effect by inducing an immunoinhibitory milieu. To make our study even more translational, testing different concentrations of rhIL-15 and understanding the optimal concentration leading to the induction of an anti-inflammatory milieu under IFNγ-induced inflammatory conditions may be required. Additionally, gene expression analysis for anti-apoptotic factors like Bcl-2 or Bcl-xL may help clarify the mechanism behind IL-15-induced protection against M2 MAC apoptosis. However, given the number of MACs in human skin, it would be interesting to study this anti-apoptotic effect by histology and/or FACs analyses. Furthermore, our data have been generated only in female donors’ skin, and investigating sex-based differences in the context of IL15 impacts on MACs may be important. Indeed, a study found that IL-15–mediated CD4+ T cell activation was higher in female donors than in male donors [44].

Though IL-15Rα-mediated signaling is required for this M2 CD206^+^MAC restoration, it does not appear to play a significant role in promoting IL-10 production by M2 CD206^+^MACs. This aligns with the previously documented role of IL-15 signaling in promoting IL-10 production from NK cells [45] mediated through the STAT3 pathway [46] which is associated with IL-15Rβ/JAK1 and not IL-15Rα. If this IL-15Rβ/JAK1 signaling is similarly responsible for IL-15′s promotion of IL-10 production by M2 CD206^+^MACs as well, then continual study of this pathway is crucial to ensure that commonly prescribed JAK1/3 inhibitors are not abrogating this anti-inflammatory, protective effect through inhibiting IL-15Rβ/γc signaling [43,45].

Thus, it deserves to be explored whether, under defined conditions, rhIL-15 or small peptide IL-15Rα agonists [43,45,47] may be beneficial in the management of IFNγ-driven inflammatory immunodermatoses, where pro-inflammatory dermal M1 ΜACs play a key role in the pathogenesis, such as psoriasis [12,14,35,48] or atopic dermatitis [14,15,48] and may be counterbalanced by therapeutically enhancing the activities of resident anti-inflammatory M2 MACs [3,49]. Indeed, preliminary studies have as yet shown that rhIL-15 and IL-15 superagonists can reduce skin inflammation in lesional human psoriasis skin [35] and in an IL-15^−/−^ mouse model of “atopic dermatitis.” [34] Incidentally, our pilot study also introduces IFNγ as an apoptosis-inducing cytokine in native dermal CD68^+^ and CD206^+^ MACs in human skin, which may contribute to the perpetuation of chronic skin inflammation by reducing the pool of immunoinhibitory dermal MACs. The demonstrated ability of rhIL-15 to counteract this effect of IFNγ further encourages one to interrogate the underestimated anti-inflammatory activities of this multi-faceted cytokine [27,47,48,50].

## 4. Materials and Methods

### 4.1. Human Eyelid Skin Samples

Full-thickness eyelid skin from 7 healthy female patients (mean age: 60.7 years old; range: 41–72 years old) undergoing blepharoplasty were utilized since its exceptionally thin and almost gel-like dermis, greatly facilitates cytokine penetration and thus access to dermal MACs under organ culture conditions [51,52]. Four of the 7 donors were used in our first organ culture experiment (see Section 4.2), and three of the 7 donors were used in the second organ culture experiment (see Section 4.3) with multiple independent skin fragments derived from each individual donor. The use of anonymized, otherwise discarded human tissue is considered non-human subject research and is exempt from IRB approval under 45 CFR46.101.2/University of Miami Miller School of Medicine.

### 4.2. Eyelid Skin Organ Culture

4 mm punch fragments of full-thickness eyelid skin were organ-cultured in a supplemented, serum-free medium that sustains human skin viability, promotes immune cell survival ex vivo [6,53] and has been utilized in established ex vivo models of chronic inflammatory conditions [6,23,54]. We have utilized organ-cultured healthy human eyelid skin in the presence of IFNγ as this offers an excellent model for interrogating human dermal MACs within their native mesenchymal tissue habitat ex vivo. Our culture medium consists of William’s E and RPMI medium (1:1) (Gibco, Thermo Fisher Scientific, Waltham, MA, USA) supplemented with 1% penicillin/streptomycin mix (Gibco, Thermo Fisher Scientific, Waltham, MA, USA), 2 mmol/L L-glutamine (Gibco, Thermo Fisher Scientific, Waltham, MA, USA), 10 ng/mL hydrocortisone (Sigma-Aldrich, Merck Millipore, Burlington, MA, USA), and 10 μg/mL insulin (Sigma-Aldrich, Merck Millipore, Burlington, MA, USA) [6,54]. The skin fragments were randomly assigned to four groups: Vehicle (5% PBS), rhIL-15 (100 ng/mL rhIL-15, Bio-Techne, Minneapolis, MN, USA), Inc., 247-ILB-025), IFNγ-induced inflammation [100 international units (IU)/mL IFNγ, Peprotech, Cranbury, NJ, USA], or rhIL-15 in IFNγ-induced inflammation (100 IU/mL IFNγ for 48 h followed by 100 ng/mL rhIL-15 for 4 days) [23,55]. Concerning the concentration of IFNγ, we used the concentration of 100 IU/mL (corresponding to 50 ng/mL) as per previous published studies [56,57] using IFNγ to mimic inflammatory conditions in ex vivo human skin organ culture (note that 10x higher concentration difference is usually used between in vitro and ex vivo). Furthermore, our concentration of IFNγ is also lower compared to previous in vitro studies using 500 U/mL [58] or 100 ng/mL [59] or to stimulate primary keratinocytes in an in vivo study, injecting intradermally 1 × 10^6^ IU of IFNγ to induce an inflammatory state in healthy patients [60]. Organ culture was performed for 6 days at 37 °C, under 5% CO_2_ [6,54] with medium changes every other day. After 6 days of culture, skin punches were fixed in 4% paraformaldehyde, washed three times in PBS, and incubated overnight with 10% sucrose in PBS at 4 °C [55] before cryoembedding, snap freezing in liquid nitrogen, and cutting into 7 µm cryosections (Cryostar NX50 Cryostat, Thermo Fisher Scientific, Waltham, MA, USA).

### 4.3. IL-15Rα Silencing Ex Vivo

Additionally, 2 mm full-thickness human eyelid skin fragments were transfected with a small interfering RNA (siRNA) probe (10 nmol ON-TARGETplus Human IL-15Rα (3601) siRNA-SMARTpool, [siIL-15Rα, Horizon Discovery Limited., Cambridge, UK, cat. L-007935-00-0010] or 5 nmol non-targeting oligos [NTO] [ON-TARGETplus Non-targeting Pool, Horizon Discovery Ltd., Cambridge, UK, cat. D-001810-10-05]) using Lipofectamine™ RNAiMAX (Life Technologies, Carlsbad, CA, USA) [23,41]. 1 µM of small interfering RNA (siRNA) against IL-15Rα or non-targeting oligos (NTO) was added 24 h after performing 2 mm diameter skin fragments for 48 h. Three full-thickness human skin fragments were collected and frozen for RNA extraction and subsequent quantitative reverse transcription-polymerase chain reaction (qRT-PCR). 100 ng/mL of recombinant human (rh) IL-15 was added to the culture 24 h after siRNA or NTO administration [23,41]. The following day, the medium was replaced and the NTO and siIL-15Rα transfected human eyelid skin punches were treated with either 100 ng/mL rhIL-15 or 100 ng/mL rhIL-15+100 IU/mL IFNγ. 48 h later, the human eyelid skin fragments were fixed for 3 h in 4% paraformaldehyde in phosphate-buffered saline (PBS) and kept overnight in 10% sucrose at 4 °C. Finally, the fixed punches were OCT-embedded, snap-frozen in liquid nitrogen, and cut into 7 µm sections for (immuno-)histology using CryoStar NX50 cryostat (Thermo Fisher Scientific, Waltham, MA, USA).

### 4.4. Quantitative Reverse Transcription-Polymerase Chain Reaction

Total RNA was isolated from full-thickness human eyelid skin using the PicoPure™ RNA Isolation Kit (Applied Biosystems–Thermo Fisher Scientific, Waltham, MA, USA) following the manufacturer’s instructions [23,41]. RNA purity and concentrations were determined using the Nanodrop ND-1000 assay (Thermo Fisher Scientific, Waltham, MA, USA). Reverse transcription of the RNA into cDNA was performed using the TetrocDNA Synthesis Kit (Bioline-Meridian Bioscience, London, UK), according to the manufacturer’s instructions. RNA concentrations were adjusted between 50 and 500 nM to have the same amount of RNA among the same donor to allow further quantification and comparison between the samples after quantitative reverse transcription-polymerase chain reaction (qRT-PCR). Normalization was performed using the housekeeping gene GAPDH. qRT-PCR was run in triplicate using TaqMan Fast Advanced Master Mix and Gene Expression Assay probes (Id: Hs02786624_g1 for GAPDH, and Hs00542602_g1 for IL-15Rα, Thermo Fisher Scientific, Waltham, MA, USA) on a CFX Duet Real-Time PCR System (BioRad, Hercules, CA, USA). The BioRad CFX Duet Real-Time PCR software collected and stored real-time quantification plots and Ct values. The amount of the transcripts was normalized to those of the housekeeping gene (GAPDH) using the ΔΔCT method [41,61,62,63].

### 4.5. Immunofluorescence Microscopy and Quantitative Immunohistomorphometry

The number of specifically immunoreactive cells with a DAPI^+^ nuclei were analyzed within the papillary dermis in a standardized reference area of 200 µm from the basement membrane of the epidermis on non-consecutive sections using ImageJ software version 2.9.0 (NIH, Bethesda, MD, USA) and our previously described qIHM methods [6,23]. IL-15Rα expression was analyzed by measuring the average intensity level of ten selected IL-15Rα-positive cells in the standardized reference area.

Dermal macrophages were identified with CD68, a pan-macrophage marker, using the BZ-X800 Fluorescence Microscope (Keyence Corporation, Osaka, Japan) [49]. M2 MACs were identified with the CD206 marker alone or in combination with CD68 [37,64] while M1 MACs were identified with the CD86 marker in combination with CD68. Immunoinhibitory M2 MACs were further identified with IL-10 in addition to CD206 [65]. To detect apoptotic cells, the TdT-mediated dUTP-biotin nick end labeling (TUNEL) [66,67] from the ApopTag^®^ Fluorescein In Situ Apoptosis Detection Kit (Merck Millipore, Burlington, MA, USA) was utilized following the manufacturer’s protocol.

Cryosections from 4% paraformaldehyde-fixed punches were treated with 5% bovine serum albumin (BSA) in PBS or not (Table 1) and incubated overnight with the respective primary antibodies (Table 1), which were diluted in PBS containing either 1% BSA or 2% goat serum at 4 °C. The cryosections were consecutively washed three times for 5 min with PBS then incubated with the corresponding fluorescently labeled secondary antibody (Table 1) at room temperature (RT) for 45 min in a humid chamber. Lastly, the cryosections were washed three times in PBS, mounted in Fluoromount-G/DAPI (Electron Microscopy Sciences, Hatfield, PA, USA), and stored at –20 °C until immunofluorescence imaging. To confirm that using two primary antibodies, rabbit-derived, did not show any cross-reactivity and show the expected specificity, we previously tested our protocol. We first stained the slides for CD68, applied the primary antibody overnight, and then incubated the slides with a saturation concentration of secondary antibody (goat anti-rabbit Alexa fluor 555). Then, we did a 30 min blocking step with 10% BSA, before applying the primary and secondary antibody for CD206.

As shown in the representative pictures (Figure 5c), some CD68^+^/CD206^−^ and CD68^−^CD206^+^ cells were identified, indicating the absence of cross-reaction between both primary antibodies. The absence of cross-reactivity was tested by staining one section with the primary and secondary antibodies for CD68 and only the secondary antibody for CD206. This showed no positive signal in the green channel (Alexa Fluor 488 was used in this case as a secondary antibody for CD206), indicating the efficiency of the blocking step after the secondary antibody for CD68 and the primary antibody for CD206 steps. The similarity of the immunostaining results to previously published, rigorously controlled antigen expression patterns was confirmed as immunostaining specificity [6,37,62,64].

The number of CD68^+^, CD206^+^ or CD68^+^CD206^+^ cells was counted in a defined reference area in the papillary dermis (200 μm below the dermal-epidermal junction) and expressed as cell number per mm^2^ (Figure 1a, Figure 2a and Figure 5a) [6]. The percentage of CD68^+^CD206^+^ was calculated by counting the number of CD68^+^CD206^+^ cells in the reference area in the papillary dermis among the total number of CD68^+^ cells in the same area (Figure 5b).

Quantitative immunohistomorphometry (qIHM) data for each donor were normalized to the vehicle or NTO (set as 1) to stratify inter-individual variability between donors. Single- or double-positive cells in close proximity (>50 µm) to hair follicles were excluded, given that perifollicular dermal sheath MACs may underlie distinct, hair follicle-regulated controls.

### 4.6. Statistical Analysis

All data are expressed as fold change in mean or mean ± SEM and were analyzed by unpaired Student’s *t*-test and Mann–Whitney U-test, two-tailed for data following Gaussian and non-Gaussian distribution, respectively (GraphPad 9 Prism, GraphPad Software, San Diego, CA, USA) after performing d’Agostino and Pearson omnibus normality test. *p* < 0.05 was regarded as significant. The number of eyelid skin sections and donors analyzed for each data set are stated in the corresponding figure legends.

## Figures and Tables

**Table 1:** List of all primary and secondary antibodies utilized. Abbreviations: CD68, cluster of differentiation 68; CD86, cluster of differentiation 86; CD206, cluster of differentiation 206; IL-10, interleukin-10; IL-15Rα, interleukin-15 receptor alpha; BSA, bovine serum albumin; TBS, tris-buffered saline; PBS, phosphate-buffered saline; N/A, not applicable.

Antigen	Blocking	Primary Antibody	Secondary Antibody	References
CD68	N/A	Rabbit anti-human CD68 [EPR23917-164] Abcam, ab213363 1:50	Goat anti-rabbit IgG-Alexa Fluor^®^ 555 Life Technology 1:400	[6]
CD86	5% BSA in TBS	Mouse anti-human CD86 Novus Biologicals, NBP2-25208 1:50	Goat anti-mouse IgG FITC Jackson ImmunoResearch 1:200	[62]
CD206	N/A	Rabbit anti-mannose receptor CD206 Abcam, ab64693 1:50	Goat anti-rabbit IgG FITC Life Technology 1:200	[38,62,65]
IL-10	N/A	Mouse anti-human IL-10 R&D systems, MAB217 1:50	Goat anti-mouse- Rhodamine Jackson ImmunoResearch 1:200	[23,36]
IL-15Rα	5% BSA in PBS	Mouse anti-IL-15Rα Abcam, ab91270, clone JM7A4 1:100	Goat anti-mouse Alexa Fluor^®^ 594 Life Technology 1:400	[23,41]

## Data Availability

The data generated during and/or analyzed during the current study are available from the corresponding author upon reasonable request. No large datasets were generated or analyzed during the current study.

## Abbreviations

The following abbreviations are used in this manuscript:

CD68	Cluster of differentiation 68
CD86	Cluster of differentiation 86
CD206	Cluster of differentiation 206
IL-10	Interleukin-10
IL-15	Interleukin-15
IL-15Rα	Interleukin-15 receptor alpha
BSA	Bovine serum albumin
TBS	Tris-buffered saline
PBS	Phosphate-buffered saline
IFNγ	Interferon gamma
SEM	Standard error of the mean
MAC	Macrophage
qIHM	Quantitative immunohistomorphometry
iNKT10	IL-10-secreting inhibitory natural killer T
siIL-15Rα	Small interfering RNA interleukin-15 receptor alpha
DAPI	4′,6-diamidino-2-phenylindole
TUNEL	TdT-mediated dUTP-biotin nick end labeling
N/A	Not applicable
